# Aberrant Intracellular pH Regulation Limiting Glyceraldehyde-3-Phosphate Dehydrogenase Activity in the Glucose-Sensitive Yeast *tps1*Δ Mutant

**DOI:** 10.1128/mBio.02199-20

**Published:** 2020-10-27

**Authors:** Frederik Van Leemputte, Ward Vanthienen, Stefanie Wijnants, Griet Van Zeebroeck, Johan M. Thevelein

**Affiliations:** a Laboratory of Molecular Cell Biology, Institute of Botany and Microbiology, Department of Biology, KU Leuven, Leuven-Heverlee, Flanders, Belgium; b Center for Microbiology, VIB, Leuven-Heverlee, Flanders, Belgium; Yonsei University

**Keywords:** glycolysis, intracellular pH, glyceraldehyde-3-phosphate dehydrogenase, trehalose-6-phosphate synthase, *Saccharomyces cerevisiae*, glucose metabolism, *TPS1*

## Abstract

Whereas the yeast Saccharomyces cerevisiae shows great preference for glucose as a carbon source, a deletion mutant in trehalose-6-phosphate synthase, *tps1*Δ, is highly sensitive to even a few millimolar glucose, which triggers apoptosis and cell death. Glucose addition to *tps1*Δ cells causes deregulation of glycolysis with hyperaccumulation of metabolites upstream and depletion downstream of glyceraldehyde-3-phosphate dehydrogenase (GAPDH). The apparent metabolic barrier at the level of GAPDH has been difficult to explain. We show that GAPDH isozyme deletion, especially Tdh3, further aggravates glucose sensitivity and metabolic deregulation of *tps1*Δ cells, but overexpression does not rescue glucose sensitivity. GAPDH has an unusually high pH optimum of 8.0 to 8.5, which is not altered by *tps1*Δ. Whereas glucose causes short, transient intracellular acidification in wild-type cells, in *tps1*Δ cells, it causes permanent intracellular acidification. The *hxk2*Δ and *snf1*Δ suppressors of *tps1*Δ restore the transient acidification. These results suggest that GAPDH activity in the *tps1*Δ mutant may be compromised by the persistently low intracellular pH. Addition of NH_4_Cl together with glucose at high extracellular pH to *tps1*Δ cells abolishes the pH drop and reduces glucose-6-phosphate (Glu6P) and fructose-1,6-bisphosphate (Fru1,6bisP) hyperaccumulation. It also reduces the glucose uptake rate, but a similar reduction in glucose uptake rate in a *tps1*Δ *hxt2,4,5,6,7*Δ strain does not prevent glucose sensitivity and Fru1,6bisP hyperaccumulation. Hence, our results suggest that the glucose-induced intracellular acidification in *tps1*Δ cells may explain, at least in part, the apparent glycolytic bottleneck at GAPDH but does not appear to fully explain the extreme glucose sensitivity of the *tps1*Δ mutant.

## Abstract (precis)

Glucose catabolism is the backbone of metabolism in most organisms. In spite of numerous studies and extensive knowledge, major controls on glycolysis and its connections to the other metabolic pathways remain to be discovered. A striking example is provided by the extreme glucose sensitivity of the yeast *tps1*Δ mutant, which undergoes apoptosis in the presence of just a few millimolar glucose. Previous work has shown that the conspicuous glucose-induced hyperaccumulation of the glycolytic metabolite fructose-1,6-bisphosphate (Fru1,6bisP) in *tps1*Δ cells triggers apoptosis through activation of the Ras-cAMP-protein kinase A (PKA) signaling pathway. However, the molecular cause of this Fru1,6bisP hyperaccumulation has remained unclear. We now provide evidence that the persistent drop in intracellular pH upon glucose addition to *tps1*Δ cells likely compromises the activity of glyceraldehyde-3-phosphate dehydrogenase (GAPDH), a major glycolytic enzyme downstream of Fru1,6bisP, due to its unusually high pH optimum. Our work highlights the potential importance of intracellular pH fluctuations for control of major metabolic pathways.

## INTRODUCTION

Identification of the mutant allele in several glucose-negative strains of the yeast Saccharomyces cerevisiae and cloning of the trehalose-6-phosphate (Tre6P) synthase gene unexpectedly converged on the same gene, *TPS1* (1–8). Trehalose-6-phosphate synthase uses glucose-6-phosphate (Glu6P) and UDP-glucose (UDPG) as the substrates to catalyze the first step in trehalose biosynthesis, followed by *TPS2*-encoded trehalose-6-phosphate phosphatase, which dephosphorylates Tre6P into trehalose (9). The *tps1* mutants, but not the *tps2* mutants, are unable to grow on glucose or other rapidly fermentable sugars. The addition of glucose to *tps1* cells causes hyperaccumulation of glycolytic metabolites upstream of glyceraldehyde-3-phosphate dehydrogenase (GAPDH) and depletion of metabolites downstream of GAPDH (1, 5, 10–16). Glu6P, fructose-6-phosphate, and especially fructose-1,6-bisphosphate (Fru1,6bisP) hyperaccumulate, while dihydroxyacetone phosphate and glyceraldehyde-3-phosphate (GA3P) increase to a lesser extent, likely because the equilibrium of the aldolase reaction is located toward Fru1,6bisP. Hence, the extent of Fru1,6bisP hyperaccumulation is a good indicator of the apparent glycolytic bottleneck at the level of GAPDH in *tps1*Δ cells. This is consistent with data on the control of the overactive glycolytic flux in cancer cells, where GAPDH was also identified as the rate-limiting step in the pathway and the level of Fru1,6bisP found to be predictive for the rate and control of glycolytic flux at GAPDH (17).

Inactivation of hexokinase 2 (Hxk2) restores normal growth of *tps1* mutants on glucose (13), and *tps1* mutants also show normal growth on galactose, for which the catabolism bypasses the hexokinase step in glycolysis (1–6, 8). These observations suggested that unregulated hexokinase activity was responsible for the growth defect of *tps1* mutants on glucose. This was confirmed by the discovery that Tre6P is a potent competitive inhibitor of hexokinase (18). On the other hand, the extreme sensitivity of *tps1* mutants to just a few millimolar glucose in the presence of 100 mM galactose (14), the partial complementation by the Escherichia coli TPS-encoding *otsA* (19), the discrepancies between Tre6P levels and the glycolytic deregulation (20), and the absence of a glucose growth defect in yeast cells expressing Tre6P-insensitive hexokinase from Schizosaccharomyces pombe (21) suggest that the absence of Tre6P inhibition of hexokinase is not the sole cause of the glucose growth defect and deregulation of glycolysis. Whereas the suppression of the *tps1*Δ growth defect on glucose by reduction of hexokinase activity suggests a plausible underlying mechanism, several other suppressors of *tps1*Δ have been identified, for which the action mechanism remains unclear: the overexpression of Mig1 (2), enhanced glycerol production (22, 23), inactivation of specific components of the electron transport chain (24), inhibition of respiration by antimycin (2, 24), and inactivation of Snf1 (25). In general, however, many of these suppressors seem to act by deviating the high levels of sugar phosphates into glycerol production, thus providing ample NAD^+^ and P_i_ for GAPDH activity but, at the same time, also preventing the induction of apoptosis by hyperaccumulation of Fru1,6bisP.

GAPDH catalyzes the first step in lower glycolysis by converting glyceraldehyde-3-phosphate (GA3P) to 1,3-bisphosphoglycerate (1,3bisPG) at the expense of P_i_ and reduction of NAD^+^ (26). It has remained unclear why glycolysis stalls at the level of GAPDH in *tps1* mutants. One possible explanation is that GAPDH uses free phosphate (P_i_) as a substrate and that the hyperaccumulation of sugar phosphates depletes the P_i_ level to such an extent that it compromises *in vivo* GAPDH activity (1, 15). Suppression of the *tps1*Δ growth defect on glucose by stimulation of glycerol biosynthesis is consistent with this explanation (27), since it recovers free phosphate but, on the other hand, also reduces sugar phosphate hyperaccumulation. The recent discovery that Fru1,6bisP is a potent stimulator of reactive oxygen species (ROS) formation and apoptosis in the *tps1*Δ strain in the presence of glucose through activation of the Ras-cAMP-protein kinase A (PKA) pathway therefore provides an alternative explanation for the suppression of *tps1*Δ by stimulation of glycerol production (12). This mechanism is supported by a recent report that the antioxidant *N*-acetylcysteine restores growth of the *tps1*Δ strain on low glucose (28). In addition, increasing the P_i_ level by itself does not revert the inefficiency of permeabilized *tps1*Δ spheroplasts to ferment glucose into ethanol (29), and extracellular addition of up to 50 mM P_i_ and/or the overexpression of P_i_ transporters failed to rescue the *tps1*Δ strain on glucose medium (22). This suggests that other mechanisms are involved that constrict glycolytic flux through GAPDH. Experimental observations also point toward GAPDH as an important bottleneck in the overactive glycolytic flux or Warburg effect in cancer cells (17, 30). It has been reported that the catalytic cysteine of GAPDH in mammalian cells is sensitive to oxidation by reactive oxygen species (ROS) and possibly helps to rewire flux toward the oxidative pentose phosphate pathway to generate more reducing power in the form of NADPH (31, 32).

In S. cerevisiae, GAPDH is a homotetramer composed of three isoforms, encoded by *TDH1*, *TDH2*, and *TDH3* (33). These three genes have high sequence similarity (>90%) but are differently regulated at the transcriptional level (34). During the exponential phase, *TDH3* is by far the most abundantly expressed, while *TDH2* transcription is low and *TDH1* barely expressed. As cells approach the stationary phase, expression levels of *TDH3* decline by 50%, while *TDH1* levels progressively increase and *TDH2* levels peak at the end of the exponential phase. In the stationary phase, expression levels of *TDH1* and *TDH3* are similar, while the expression of *TDH2* is low (34). The expression of *TDH1* is rapidly enhanced under various stress conditions (35–38). The catalytic activities of the three isoenzymes are different, with Tdh1 (*K_m_* = 0.86 mM; *k*_cat_ = 29.04 s^−1^) having the highest, Tdh2 intermediate (*K_m_* = 0.42 mM; *k*_cat_ = 16.22 s^−1^), and Tdh3 the lowest activity (*K_m_* = 0.25 mM; *k*_cat_ = 9.12 s^−1^), while the affinity ranks in the opposite order (39). The combined deletion of *TDH2* and *TDH3* is synthetically lethal, likely due to the low expression of *TDH1*, whereas the other double deletion strains are viable (34).

The addition of glucose to wild-type cells causes a rapid drop in the intracellular pH and ATP level, apparently due to rapid phosphorylation of the incoming glucose by hexokinase (1, 15, 40–42). However, in wild-type cells, this drop in intracellular pH and ATP level is only transient, and its recovery is faster under aerobic conditions than anaerobic conditions (41). On the other hand, in *tps1*Δ cells, the drop in the ATP level and the intracellular acidification are permanent (1, 23, 42). The consumption of ATP in the initiation of glycolysis and the generation of extra ATP downstream in glycolysis are no longer balanced, and the overactive sugar phosphorylation results in an ATP trap (43).

In the present study, we aim to understand the mechanism behind the glycolytic bottleneck at the level of GAPDH upon the addition of glucose to *tps1*Δ cells. We have explored the possibility that the persistent intracellular acidification observed after the addition of glucose to *tps1*Δ cells might be limiting glycolytic flux at the level of GAPDH because of its unusually high pH optimum. We provide evidence that the prevention of the glucose-induced pH drop partially suppresses sugar phosphate hyperaccumulation and metabolic defects in *tps1*Δ cells. Our results highlight the importance of intracellular pH in the control of glycolytic flux at the level of GAPDH and reveal novel roles for Tps1 in the control of glycolysis beyond its role in limiting hexokinase activity.

## RESULTS

### Influence of single GAPDH isoforms on the *tps1*Δ glucose growth defect.

**FIG 1:**
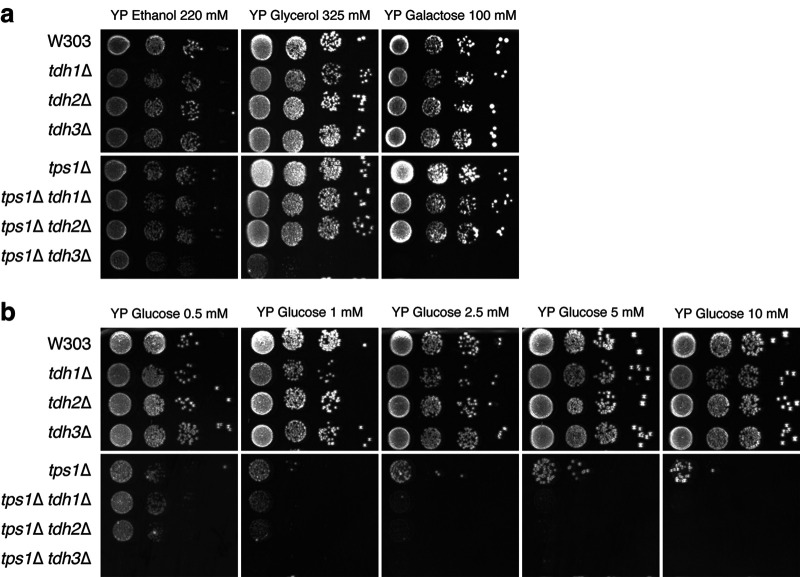
Single *TDH* isoform deletions increase the glucose sensitivity of the *tps1*Δ strain but do not affect growth on glucose of the wild-type strain. Wild-type and *tps1*Δ cells and their respective *TDH* single-deletion derivatives were spotted on YP agar in serial 5-fold dilutions after pregrowth on 3% glycerol. Cells were spotted onto plates containing 220 mM ethanol, 325 mM glycerol, or 100 mM galactose (a) or increasing concentrations of glucose: 0.5, 1, 2.5, 5 or 10 mM (b). Pictures were taken after 3 days.

To investigate whether a specific GAPDH isoform played a significant role in the glucose sensitivity of the *tps1*Δ strain, single *TDH* deletions were introduced in wild-type and *tps1*Δ strains. Subsequently a spot test was performed to test growth of these strains on different respiratory carbon sources (ethanol, glycerol, and galactose) as well as different glucose concentrations (Fig. 1). Consistent with the literature, the deletion of single GAPDH isoforms did not affect growth in the W303 wild-type background on any of the carbon sources tested (Fig. 1a and b). In the *tps1*Δ strain, on the other hand, the additional deletion of *TDH1* or *TDH2* worsened the *tps1*Δ growth defect at low glucose concentrations (Fig. 1b), while growth on the respiratory carbon sources was unaffected (Fig. 1a). The deletion of *TDH3* in the *tps1*Δ strain, on the other hand, completely abolished growth at low glucose concentrations, down to 0.5 mM, with which the *tps1*Δ strain still grew to some extent. Since Tdh3 is responsible for most of the GAPDH activity when yeast is growing with glucose (39), this aggravated glucose sensitivity suggests that the deletion of *TDH3* increases the metabolic bottleneck at the level of GAPDH. Unexpectedly, however, the additional deletion of *TDH3* in the *tps1*Δ strain also completely abolished growth on galactose and allowed only residual growth on glycerol. In the wild-type strain, the deletion of *TDH3* did not cause any effect for growth on galactose or glycerol. Growth on ethanol, for which *TDH3* is not required, was not affected in any strain. These results indicate a novel interaction between Tps1 and the metabolism of galactose and glycerol, since hexokinase, the only known target of Tre6P, the product of Tps1, is not involved in the metabolism of galactose and glycerol.

**FIG 2:**
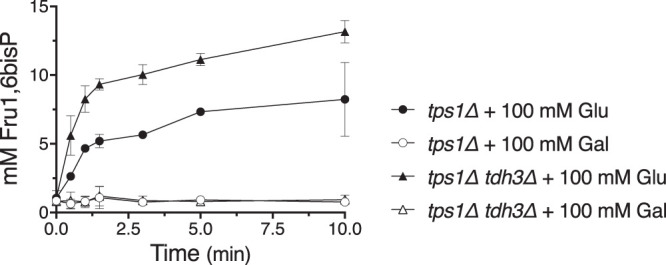
The deletion of *TDH3* enhances Fru1,6bisP accumulation in the *tps1*Δ strain after the addition of glucose but not after addition of galactose. Fru1,6bisP accumulation was measured after addition of 100 mM glucose (closed symbols) or galactose (open symbols) to cells of the *tps1*Δ strain (circles) or *tps1*Δ *tdh3*Δ strain (triangles). Cells were pregrown on complete synthetic medium with 3% glycerol as carbon source.

To know if the reduced growth capacity of the *tps1*Δ *tdh3*Δ strain on galactose medium involves a similar bottleneck in the glycolytic pathway as that observed on glucose (12), we measured intracellular Fru1,6bisP levels after addition of 100 mM galactose or 100 mM glucose to glycerol-grown *tps1*Δ *tdh3*Δ cells (Fig. 2). Clearly, addition of galactose did not cause Fru1,6bisP hyperaccumulation in the *tps1*Δ *tdh3*Δ strain compared to that in the *tps1*Δ strain. In contrast, the deletion of *TDH3* in the *tps1Δ* strain caused a further increase in Fru1,6bisP hyperaccumulation compared to that in the *tps1*Δ strain. This result indicates that the extent of Fru1,6bisP hyperaccumulation is inversely correlated with the residual GAPDH activity in the *tps1*Δ strain and that GAPDH acts as a bottleneck for glycolytic flux in the *tps1*Δ strain. These results also indicate that the growth defects of the *tps1*Δ *tdh3*Δ strain on glucose and galactose are apparently caused by different mechanisms and that the growth defect on galactose is not due to insufficient GAPDH activity.

**FIG 3:**
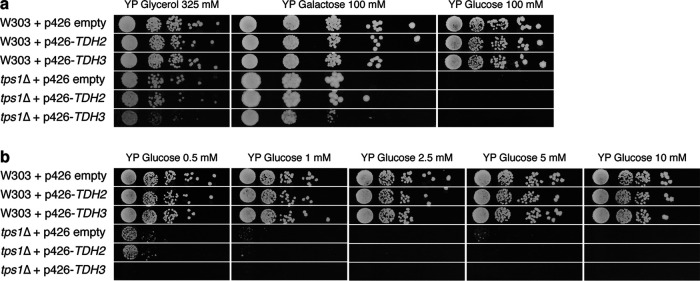
The overexpression of *TDH2* or *TDH3* does not rescue the glucose growth defect of the *tps1*Δ strain. Plasmid-transformed cells of wild-type and *tps1*Δ strains were spotted in serial 5-fold dilutions on YP agar supplemented with 325 mM glycerol, 100 mM galactose, or 100 mM glucose (a) or different concentrations of glucose: 0.5, 1, 2.5, 5 or 10 mM (b). *TDH*2 and *TDH3* were overexpressed from the p426 multicopy plasmid behind the strong constitutive *TEF1* promoter. Strains containing the p426 plasmid without an insert served as controls. Pictures were taken after 3 days.

To evaluate whether the apparent bottleneck of glycolytic flux in the *tps1*Δ strain at the level of GAPDH is merely due to insufficient catalytic activity, we next investigated whether the overexpression of GAPDH isoforms could overcome the *tps1*Δ growth defect on glucose. For this purpose, the multicopy p426 plasmid was used for the overexpression of the GAPDH isogenes *TDH2* and *TDH3* using the constitutive *TEF1* promoter. Since *TDH1* is mostly expressed in stationary-phase cells (35), we did not select this isogene for overexpression. Glycerol-grown precultures were spotted onto rich solid nutrient medium containing different carbon sources (glycerol, galactose, or glucose) (Fig. 3a) or a series of low glucose concentrations ranging between 0.5 and 10 mM (Fig. 3b). In the wild-type strain, the overexpression of *TDH2* or *TDH3* had no effect for growth on any carbon source or any concentration of glucose tested. The glucose growth defect of the *tps1*Δ strain was also not overcome by the overexpression of *TDH2* at any glucose concentration tested, while the overexpression of *TDH3* even aggravated the glucose-sensitive phenotype.

### GAPDH activity is highly sensitive to changes in cytosolic pH.

**FIG 4:**
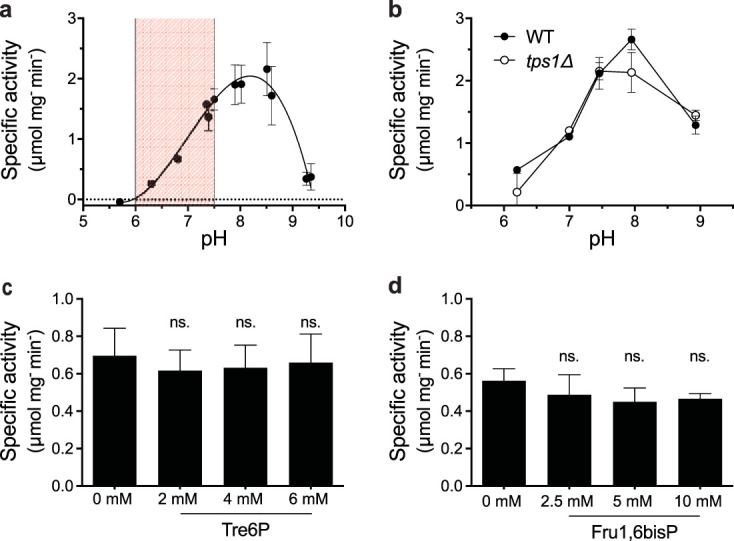
The *in situ* GAPDH activity is strongly compromised at low pH and not affected by Tre6P or Fru1,6bisP. GAPDH activity was measured in permeabilized spheroplasts of wild-type cells studied over a wide pH range (highlighted: physiological pH range from 6 to 7.5) (a) and wild-type compared to *tps1*Δ cells (b). GAPDH activity was measured in permeabilized wild-type cells at pH 6.8 in the absence or presence of increasing concentrations of Tre6P (2, 4, or 6 mM) (c) and Fru1,6bisP (2.5, 5, or 10 mM) (d). Statistical analysis was performed by one-way analysis of variance (ANOVA) with Dunnett’s multiple-comparison test. No significant difference was observed between the samples with and without Tre6P or Fru1,6bisP. ns, not significant, *P* > 0.05.

Since the glycolytic deregulation seems to hint at compromised GAPDH activity in the *tps1*Δ strain, we first measured the pH optimum of GAPDH in permeabilized spheroplasts of wild-type cells (Fig. 4a). This *in situ* technique allows us to manipulate experimental conditions while preserving the native protein environment in the cells. While specific GAPDH activity was highest around pH 8.0, almost no activity was measured below pH 6.0. From these results, a near linear relationship of GAPDH activity with respect to pH within the physiological range between pH 6 and 7.5 becomes apparent. We also compared the pH dependency and maximal activity of GAPDH activity *in situ* between wild-type and *tps1*Δ cells (Fig. 4b). However, both strains displayed similar pH profiles and maximal activities, making it unlikely that the absence of Tps1 compromises glycolytic flux by directly influencing GAPDH activity. We also studied the influence of the two most relevant metabolites, Tre6P and Fru1,6bisP, on GAPDH activity at the physiologically relevant pH of 6.8. However, addition of neither Tre6P, nor Fru1,6bisP significantly influenced GAPDH activity in permeabilized wild-type cells, suggesting that these metabolites do not play a significant role in the deregulation of glycolysis through stimulation or inhibition of GAPDH (Fig. 4c and d).

**FIG 5:**
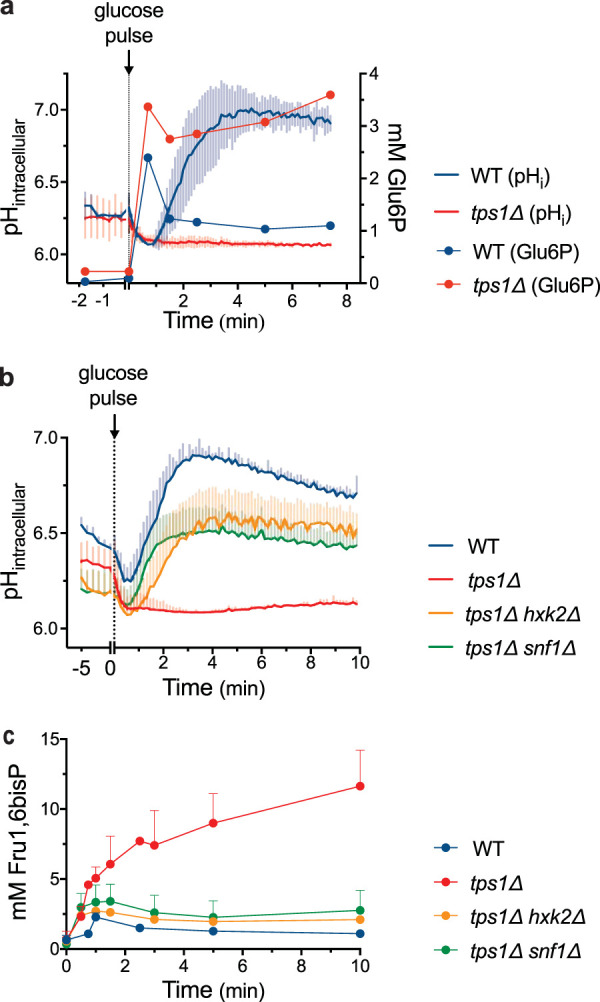
Intracellular acidification correlates with Fru1,6bisP accumulation. (a) Intracellular pH (full lines without symbols) and Glu6P levels (full lines with circles) after addition of 100 mM glucose in wild-type (blue) and *tps1*Δ (red) strains. (b) Intracellular pH profile of wild-type (blue), *tps1*Δ (red), *tps1*Δ *hxk2*Δ (yellow), and *tps1*Δ *snf1*Δ (green) strains after addition of 100 mM glucose. Standard deviations were calculated from at least 9 technical repeats per strain. (c) Fru1,6bisP levels after addition of 100 mM glucose to wild-type (blue), *tps1*Δ (red), *tps1*Δ *hxk2*Δ (yellow), and *tps1*Δ *snf1*Δ (green) strains. Cells were always pregrown on complete synthetic medium supplemented with 2% galactose.

Given the strong pH dependency and high pH optimum of GAPDH activity and the fact that glucose addition to *tps1*Δ cells is known to trigger intracellular acidification (1, 23, 42), we set out to investigate whether low cytosolic pH could be a cause of the apparent metabolic bottleneck in the glycolytic flux at the level of GAPDH after the addition of glucose to *tps1*Δ cells. To measure the intracellular pH (pH_i_) more precisely, we applied a fluorescence-based technique by expressing the cytosolic pH-sensitive green fluorescent protein (GFP) variant, pHluorin, in the cells of interest (44). We have compared the pH_i_ profiles following glucose addition to pHluorin-expressing cells of wild-type, *tps1*Δ, and the *tps1*Δ suppressor strains *tps1*Δ *hxk2*Δ and *tps1*Δ *snf1*Δ. As previously reported (1, 15, 40–42), the pH_i_ of wild-type cells transiently dropped and rapidly recovered to approximately neutral pH_i_ in response to glucose addition, while *tps1*Δ cells were unable to recover from the initial pH drop and showed persistent intracellular acidification (Fig. 5a). The transient or persistent drop in pH_i_ coincided with a transient or persistent increase in the Glu6P level, respectively. Interestingly, the initial Glu6P peak (45 s after glucose addition) in wild-type cells corresponded to a pH_i_ of approximately 6.0, at which specific GAPDH activity measured *in vitro* is minimal. Moreover, the *tps1*Δ suppressor strains, *tps1*Δ *hxk2*Δ and *tps1*Δ *snf1*Δ, clearly regained the ability to rapidly recover from the initial intracellular acidification after glucose addition, although recovery was somewhat less robust (Fig. 5b). Taken together, we observed a clear correlation between the maintenance of a proper pH_i_ and the ability to grow on glucose.

In the next step, we examined if the pH_i_ profile correlated with the apparent bottleneck in glycolytic flux at the level of GAPDH by measuring intracellular Fru1,6bisP accumulation (Fig. 5c). Indeed, among the four strains, wild type, *tps1*Δ, *tps1*Δ *hxk2*Δ, and *tps1*Δ *snf1*Δ, the capacity to recover pH_i_ after glucose addition was inversely correlated with the extent of intracellular Fru1,6bisP accumulation and thus with the apparent bottleneck at the level of GAPDH. Moreover, the transient increase in Fru1,6bisP concentration observed in wild-type cells, with a maximum at approximately 1 min after glucose addition, coincided approximately with the minimum pH_i_ value after glucose addition. Hence, our results are consistent with low pH_i_ being a limiting factor for glycolytic flux at the level of GAPDH, as inferred from Fru1,6bisP accumulation, in wild-type cells and especially in cells of the *tps1*Δ strain.

### Preventing intracellular acidification partially counteracts glycolytic deregulation.

**FIG 6:**
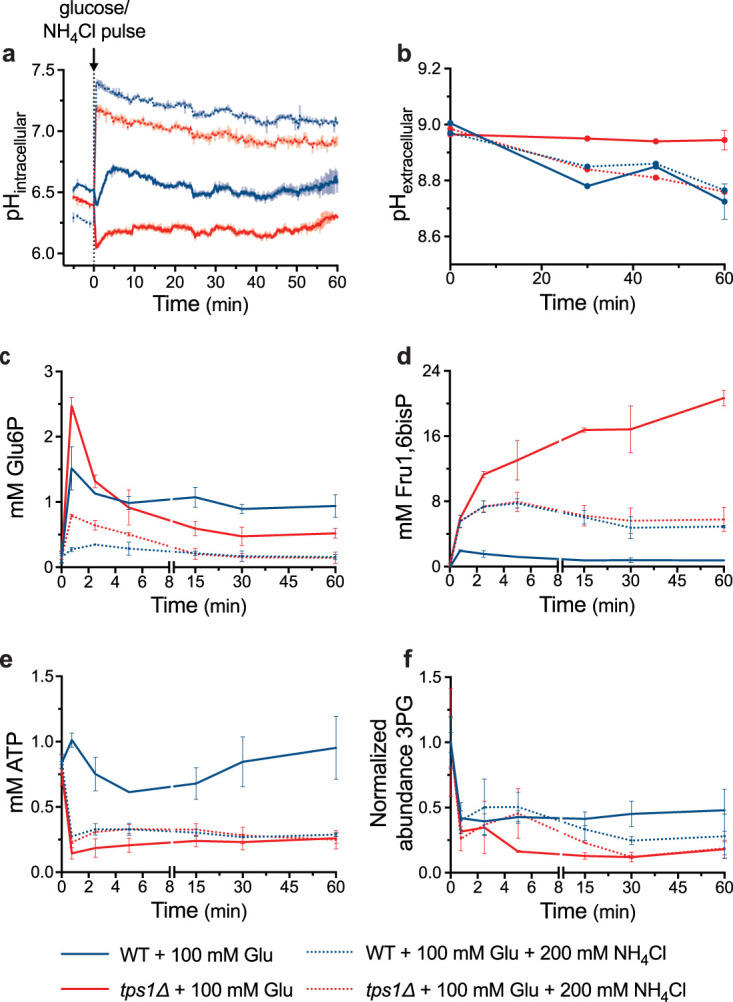
The prevention of intracellular acidification reduces Glu6P and Fru1,6bisP hyperaccumulation and partially counteracts metabolic deregulation in *tps1*Δ cells. Wild-type (blue) and *tps1*Δ (red) galactose-grown cells were resuspended in 200 mM Tris-HCl, pH 9. At time point zero, cells were provided with 100 mM glucose (full lines) or with 100 mM glucose and 200 mM NH_4_Cl (dotted lines). Graphs represent the time course profiles of intracellular pH (a), extracellular pH (b), Glu6P level (c), Fru1,6bisP level (d), ATP level (e), and normalized level of 3PG (f).

The previous experiments suggested that the pH_i_ dependency of GAPDH might, at least in part, constitute a causal factor for Fru1,6bisP hyperaccumulation in *tps1*Δ cells after glucose addition. Hence, we have applied experimental conditions to prevent the cytosolic acidification in the *tps1*Δ strain by supplementing NH_4_Cl at high extracellular pH (41). At an extracellular pH closer to the protonation equilibrium (pK*_a_*_,NH4+_ = 9.24) (45), ammonium (NH_4_^+^) shifts toward its un-ionized form, ammonia (NH_3_), which can diffuse through the plasma membrane. Once in the cell, where the pH_i_ is lower, NH_3_ converts to NH_4_^+^ taking up one proton and raising the pH_i_. By using this approach, we were able to prevent intracellular acidification in both wild-type and *tps1*Δ cells after glucose addition together with 200 mM NH_4_Cl in extracellular medium buffered with 200 mM Tris-HCl at pH 8, 8.5, or 9 (see Fig. S1a and b in the supplemental material). At an extracellular pH of 9, we were able to maintain pH_i_ between approximately 7.4 and 6.8 for 60 min in both wild-type and *tps1*Δ cells (Fig. 6a). However, at an extracellular pH of 7.9, growth was already completely prevented both in wild-type and *tps1*Δ cells (see Fig. S2a and b). This made it impossible to evaluate the rescue of growth in the *tps1*Δ strain by the prevention of intracellular acidification using this approach. On the other hand, we were able to determine the effect of the maintenance of proper pH_i_ on the short-term deregulation of glycolysis in *tps1*Δ cells.

10.1128/mBio.02199-20.1 FIG S1 Intracellular acidification after the addition of glucose is prevented in 200 mM Tris-HCl medium at high pH in the presence of 200 mM NH_4_Cl. Intracellular pH was monitored after addition at time point zero of 100 mM glucose with (full lines) or without (dotted lines) 200 mM NH_4_Cl. Wild-type (a) and *tps1*Δ (b) cells in 200 mM Tris-HCl medium at pH 8 (light blue), pH 8.5 (dark blue), and pH 9 (purple). Cells were grown in uracil-deficient medium with 2% galactose to retain the pHluorin plasmid. Download FIG S1, EPS file, 2.5 MB. Copyright © 2020 Van Leemputte et al. 2020 Van Leemputte et al. This content is distributed under the terms of the Creative Commons Attribution 4.0 International license.

10.1128/mBio.02199-20.2 FIG S2 High extracellular pH inhibits growth. Growth in complete synthetic medium supplemented with either 100 mM glucose (full lines) or 100 mM galactose (dotted lines) was followed over 48 h by measuring OD_595_ for wild-type (blue) and *tps1*Δ (red) cells in regular liquid medium at pH 5.5 (a) or 50 mM Tris-HCl medium at pH 8 (b). Cells were pregrown on galactose medium. Download FIG S2, EPS file, 2.7 MB. Copyright © 2020 Van Leemputte et al. 2020 Van Leemputte et al. This content is distributed under the terms of the Creative Commons Attribution 4.0 International license.

Addition of 200 mM NH_4_Cl buffered at pH 9 together with glucose effectively abolished intracellular acidification in both wild-type and *tps1*Δ cells (Fig. 6a). We next measured the extracellular pH over the course of the experiment. In spite of the high alkaline pH and the strong buffering of the medium, wild-type cells were still able to lower the pH of the medium from 9 to approximately 8.8 in 60 min after the addition of glucose, both in the presence and the absence of NH_4_Cl, which is indicative of active fermentation and proton pumping by the plasma membrane H^+^-ATPase (Fig. 6b). On the other hand, the *tps1*Δ strain was only able to lower the extracellular pH to the same extent as wild-type cells when NH_4_Cl was added together with glucose. In the absence of NH_4_Cl, there was no drop in medium pH after the addition of glucose to *tps1*Δ cells (Fig. 6b), consistent with previous reports on the absence of glucose-induced activation of plasma membrane H^+^-ATPase and a precipitous glucose-induced drop in ATP in the *tps1*Δ mutant (1, 42). This result suggests that the prevention of intracellular acidification in *tps1*Δ cells was able to restore glycolytic flux to such an extent that enough ATP could be produced for the restoration of proton pumping activity and extracellular medium acidification.

We next determined the major glycolytic metabolites under the same conditions of glucose addition with and without NH_4_Cl at extracellular pH 9 (Fig. 6c to f). The transient Glu6P accumulation was strongly reduced in the presence of NH_4_Cl (Fig. 6c). However, this was also the case in the wild-type strain, which could be attributed to decreased glucose transport because of the high extracellular pH and/or stimulation of phosphofructokinase by NH_4_^+^ (46). The latter mechanism might provide at least a partial explanation, since the glucose-induced increase in Fru1,6bisP was strongly enhanced in wild-type cells (Fig. 6d). On the other hand, the dramatic glucose-induced hyperaccumulation of Fru1,6bisP in *tps1*Δ cells was strongly reduced in the presence of NH_4_Cl and now actually overlapped with the enhanced increase of Fru1,6bisP in wild-type cells, consistent with improved glycolytic flux at the level of GAPDH (Fig. 6d). The latter was also supported by the slightly lower drop in the ATP level in *tps1*Δ cells (Fig. 6e) and higher concentration of 3-phosphoglycerate (3PG), a metabolite downstream of GAPDH in glycolysis, as measured by mass spectrometry (Fig. 6f). Glucose addition caused a much stronger drop in 3PG in *tps1*Δ cells than in wild-type cells, consistent with the bottleneck at the level of GAPDH in *tps1*Δ cells (Fig. 6f). In wild-type cells, the presence of NH_4_Cl caused a precipitous drop in the ATP level (Fig. 6e), consistent with the dramatic increase in the Fru1,6bisP level (Fig. 6d). The presence of NH_4_Cl had little effect on the 3PG level in wild-type cells in the short term but caused a drop in the longer term (Fig. 6f). These results are consistent with stimulation of glycolytic flux at the level of GAPDH in *tps1*Δ cells by counteracting the dramatic glucose-induced drop in pH_i_ and thus suggest that the persistent intracellular acidification is, at least in part, responsible for the apparent bottleneck in glycolytic flux in *tps1*Δ cells.

**FIG 7:**
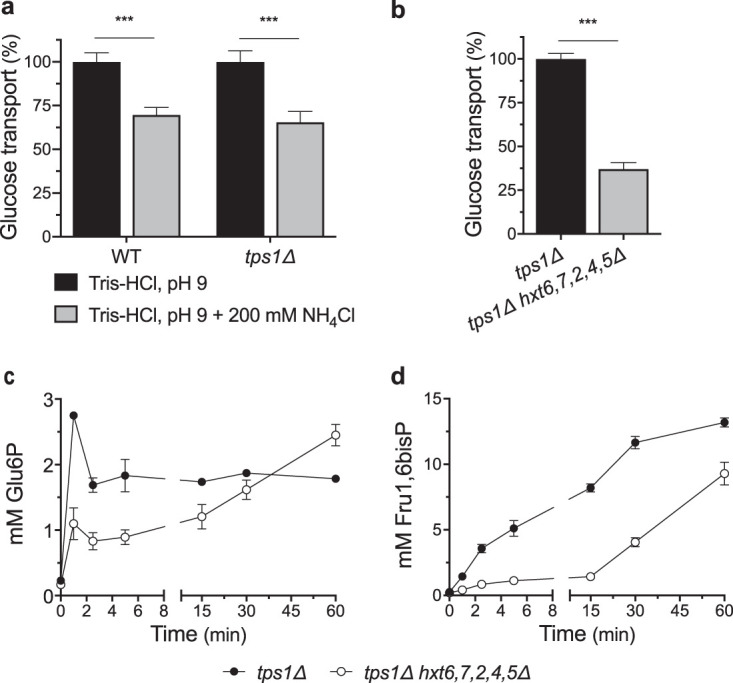
Reduced glucose transport delays but does not prevent deregulation of glycolytic flux in *tps1*Δ cells. Relative uptake rate of 100 mM glucose in wild-type and *tps1*Δ cells in 200 mM Tris-HCl, pH 9, in the absence (black bars) or presence (gray bars) of 200 mM NH_4_Cl (a) and *tps1*Δ (black bar) and *tps1*Δ *hxt6*,*7*,*2*,*4*,*5*Δ (gray bar) cells in complete synthetic medium (b). Metabolite profiles for Glu6P (c) and Fru1,6bisP (d) after addition of 100 mM glucose to galactose-grown *tps1*Δ (closed symbols) or *tps1*Δ *hxt7*,*6*,*2*,*4*,*5*Δ (open symbols) cells. Statistical analysis was performed by two-way ANOVA with Bonferroni’s correction (a) and an unpaired Student's *t* test (b). ***, *P* < 0.001.

We also evaluated the possible alternative interpretation that the addition of 200 mM NH_4_Cl at high extracellular pH of 9 in 200 mM Tris-HCl buffer reduced glucose uptake to such an extent that the glucose-induced hyperaccumulation of sugar phosphates in *tps1*Δ cells was counteracted because of reduced glucose influx. Hence, we first measured glucose uptake rate in wild-type and *tps1*Δ cells in the absence and presence of NH_4_Cl at pH 9. The uptake of 100 mM glucose was reduced 30% and 35% in wild-type and *tps1*Δ strains, respectively (Fig. 7a). To determine whether such a reduction in glucose uptake rate could counteract the glucose-induced hyperaccumulation of Glu6P and Fru1,6bisP in *tps1*Δ cells to the same extent as we observed in the presence of NH_4_Cl at pH 9, we deleted five *HXT* glucose transporter genes, *HXT6*, -*7*, -*2*, -*4*, and -*5*, in the *tps1*Δ strain. This did not restore the capacity to grow on 100 mM glucose (see Fig. S3).

10.1128/mBio.02199-20.3 FIG S3 Absence of growth on 100 mM glucose in the *tps1*Δ *hxt6,7,2,4,5*Δ strain. Cultures of the wild-type, *tps1*Δ, *hxt6,7,2,4,5*Δ, and *tps1*Δ *hxt6,7,2,4,5*Δ strains were spotted in 5-fold dilutions on YP agar with 100 mM galactose or 100 mM glucose as carbon source. Pictures were taken after three days. Download FIG S3, TIF file, 2.5 MB. Copyright © 2020 Van Leemputte et al. 2020 Van Leemputte et al. This content is distributed under the terms of the Creative Commons Attribution 4.0 International license.

The *tps1*Δ *hxt6*,*7*,*2*,*4*,*5*Δ cells were pregrown on glycerol to avoid the expression of the high-affinity galactose (and glucose) transporter *GAL2*. The *tps1*Δ *hxt6*,*7*,*2*,*4*,*5*Δ strain displayed a reduction in the glucose (100 mM) transport rate of 65% (Fig. 7b). This is approximately double the transport reduction caused by NH_4_Cl addition. The initial transient Glu6P increase was considerably reduced in the *tps1*Δ *hxt6*,*7*,*2*,*4*,*5*Δ strain compared to that in the *tps1*Δ strain, but Glu6P kept accumulating until it reached levels as high as in the *tps1*Δ strain (Fig. 7c). Similarly, Fru1,6bisP accumulation in the *tps1*Δ *hxt6*,*7*,*2*,*4*,*5*Δ strain was only delayed and not prevented (Fig. 7d). Hence, the reduction of glucose uptake of 65% in the *tps1*Δ *hxt6*,*7*,*2*,*4*,*5*Δ strain was not enough to prevent the deregulation of glycolysis, which is consistent with the inability of that strain to grow on 100 mM glucose. Since glucose uptake was reduced twice as much in the *tps1*Δ *hxt6*,*7*,*2*,*4*,*5*Δ strain compared to that under the condition with NH_4_Cl, these results make it unlikely that the reduction of glucose uptake was responsible for the diminished Glu6P and Fru1,6bisP hyperaccumulation in *tps1*Δ cells after the addition of glucose plus NH_4_Cl at high pH (Fig. 6).

## DISCUSSION

The addition of glucose to *tps1*Δ cells of the yeast S. cerevisiae causes hyperaccumulation of all glycolytic metabolites upstream and depletion of all metabolites downstream of GAPDH, suggesting that the deletion of Tps1 in some way creates a bottleneck in glycolysis at the level of GAPDH (27). Measurements of the specific activity of the glycolytic enzymes in cell extracts as well as determination of initial glucose uptake rates did not reveal significant differences between the wild-type and *tps1* strains that could explain the glycolytic bottleneck in *tps1*Δ cells (1, 11). More detailed measurements of the glucose uptake rate and the pH dependency of GAPDH in the present work have underscored the conclusion that there is no difference in the inherent activity of these two crucial components in the *tps1*Δ strain. Hence, the bottleneck appears to be due to a metabolic or regulatory problem at the level of GAPDH that is not maintained in cell extracts and is not apparent from the *V*_max_ or *K_m_* of initial glucose uptake rates in intact cells.

A possible reason for reduced GAPDH activity *in vivo* in *tps1*Δ cells might have been a direct regulatory action of Tre6P on GAPDH. This would have been in accordance with previous observations that Tre6P stimulates ethanolic fermentation in permeabilized spheroplasts of *tps1*Δ cells (29). However, we did not detect any significant influence of Tre6P on GAPDH activity in permeabilized spheroplasts (Fig. 4c), and GAPDH activity also did not show any significant deviation as a function of pH in extracts of *tps1*Δ versus that of wild-type cells (Fig. 4b). In the absence of glucose, GAPDH activity itself does not seem to be affected, since the *tps1*Δ strain displays normal growth on galactose and on nonfermentable carbon sources, for which GAPDH activity is also essential.

Since P_i_ and NAD^+^ are substrates of GAPDH, their depletion could in principle compromise *in vivo* GAPDH activity. NAD^+^ levels, however, appear to be unaffected (16), and conditions that enhance the NADH availability strongly counteract the glucose growth defect of the *tps1*Δ strain (2, 15, 24). NAD^+^ is also unlikely to be limiting, since no NAD^+^-consuming steps are present in the first part of glycolysis. Although the P_i_ level drops somewhat more upon the addition of glucose to *tps1*Δ cells compared to that for wild-type cells, the difference is small (1). The suppression of the *tps1*Δ growth defect on glucose by stimulation of glycerol biosynthesis, which causes recovery of free phosphate, initially appeared to be consistent with restriction of GAPDH activity by P_i_ limitation. However, supply of high external P_i_ levels or stimulation of P_i_ uptake by the overexpression of the Pho84 phosphate carrier does not suppress the *tps1*Δ growth defect on glucose (22, 27). In addition, the depletion of all other phosphate-containing metabolites in *tps1*Δ cells to specifically sustain the hyperaccumulation of the upstream sugar phosphates in glycolysis (16, 23) apparently provides sufficient free P_i_ for this continued buildup, which requires the maintenance of ATP production from ADP and free P_i_. This makes it unlikely that P_i_ limitation is the main cause of the apparent bottleneck at GAPDH, although it cannot be excluded that it reinforces the bottleneck.

We also explored the possible contribution of the three Tdh isoforms in the apparent GAPDH bottleneck. Although the deletion of any one of the Tdh isoforms did not affect the growth of wild-type cells, the deletion of a single isoform, and especially of the most active isoform, Tdh3, exacerbated the glucose sensitivity of the *tps1*Δ strain (Fig. 1). Moreover, the absence of Tdh3 resulted in much higher levels of Fru1,6bisP after glucose addition (Fig. 2). This reinforces the conclusion that GAPDH activity is limiting in *tps1*Δ cells and that this is a major factor for their high glucose sensitivity. On the other hand, the overexpression of Tdh isoforms did not rescue the growth defect of the *tps1*Δ strain to any extent, not even at very low glucose concentrations (Fig. 3). This indicates that the factor limiting GAPDH activity in *tps1*Δ cells overrides any increase in intrinsic GAPDH specific activity that could be established *in vivo*. An inappropriate intracellular pH could constitute such a factor, since it would severely limit the actual *in vivo* GAPDH activity, whatever the potential activity under optimal conditions.

In this work, we have explored an alternative explanation as a major cause for the apparent glycolytic bottleneck at the level of GAPDH by concentrating on the unusually high pH optimum of GAPDH and the persistent glucose-induced drop in intracellular pH in *tps1*Δ cells. When glucose is added to respiring wild-type yeast cells, there is a short transient drop in the intracellular pH, which is likely caused by the very rapid proton-producing phosphorylation of glucose (7, 41). The plasma membrane H^+^-ATPase Pma1, which is posttranslationally activated by glucose (47, 48), supports proton extrusion to the medium, while the vacuolar V-ATPase pumps protons into the vacuole, which together cause rapid recovery of the cytosolic pH_i_ to approximately neutral pH (49–51). The *tps1*Δ strain is deficient in glucose-induced activation of plasma membrane H^+^-ATPase (1, 42), and the rapid depletion of the ATP level likely compromises proton pumping activity by both Pma1 and V-ATPase (1, 42) (Fig. 6e). This explains the persistent intracellular acidification in *tps1*Δ cells (Fig. 6a) (23, 42). The limitation of proton export by the plasma membrane H^+^-ATPase likely explains the absence of glucose-induced medium acidification in *tps1*Δ cells (Fig. 6b).

Our data show that GAPDH activity is particularly sensitive to pH_i_, with a very atypical optimum at pH 8 and thus reduced activity in the physiological pH range of 6.5 to 7.5 (Fig. 4a). Between pH 6.5 and 6, the activity drops further to less than 10% of the optimal activity at pH 8. Moreover, our results indicate that, coinciding with the sugar phosphate hyperaccumulation in the *tps1*Δ strain after the addition of glucose, the intracellular pH drops to approximately 6.1 to 6.2 and does not recover, as opposed to the rapid recovery in the wild-type and *tps1*Δ suppressor strains (Fig. 5a and b). This suggests that the persistent intracellular acidification might be responsible for the apparent block of glycolysis at the level of GAPDH, causing the continuous increase in Fru1,6bisP levels in *tps1*Δ cells. In wild-type cells, the drop in intracellular pH after the addition of glucose is very transient, recovering within 1 to 2 min, and it may be supporting the transient overshoot in Glu6P and Fru1,6bisP levels that happens at that time (Fig. 5). As opposed to that in *tps1*Δ cells, the ATP level remains high in wild-type cells after the addition of glucose (Fig. 6e), consistent with rapid initiation of proper flux downstream in glycolysis and proper support of H^+^-pumping activity to establish a regular intracellular pH.

To evaluate the possible involvement of persistent intracellular acidification in *tps1*Δ cells after the addition of glucose in causing the apparent block at GAPDH and the resulting hyperaccumulation of Fru1,6bisP and other metabolic defects, we suppressed intracellular acidification by adding 200 mM NH_4_Cl at pH 9 together with glucose. This was very effective and maintained the intracellular pH at or above pH 7 for at least 60 min. The maintenance of a proper intracellular pH strongly reduced the hyperaccumulation of Fru1,6bisP, as well as the overshoot of Glu6P, and also restored the extracellular medium acidification for which proper ATP provision and proton pumping are required (Fig. 6). Hence, these results support that the maintenance of a proper intracellular pH caused the restoration of downstream glycolytic flux, at least to some extent, in the *tps1*Δ cells. This was further supported by a slightly smaller drop in the ATP level and a slight increase in 3PG levels (Fig. 6). A recent report that protonophores, such as dinitrophenol and carbonyl cyanide-4-(trifluoromethoxy)phenylhydrazone (FCCP), stimulate glucose consumption and support growth on low glucose of *tps1*Δ cells is also consistent with this explanation (28). Interestingly, in wild-type cells, the overshoot in Glu6P was also greatly suppressed when glucose was added with 200 mM NH_4_Cl at pH 9, while the increase in Fru1,6bisP was strongly elevated (Fig. 6d). The strong elevation of Fru1,6bisP might be due to the well-known stimulating effect of NH_4_^+^ on phosphofructokinase activity (46), which is consistent with the significant simultaneous drop in ATP (Fig. 6e). Since this stimulation likely happened also in *tps1*Δ cells and since the Fru1,6bisP levels in wild-type and *tps1*Δ cells coincided when glucose was added with 200 mM NH_4_Cl at pH 9, the maintenance of a proper intracellular pH appears to have largely normalized the Fru1,6bisP profile in the *tps1*Δ cells. Hence, these results further support that the persistently low pH_i_ acts as a major driver at least for the initial glycolytic bottleneck in *tps1*Δ cells, which might be aggravated further by other factors such as the stronger reduction in intracellular P_i_ than that in wild-type cells. Since both wild-type and *tps1*Δ cells were unable to grow at the high extracellular pH of 9 and since the addition of 200 mM NH_4_Cl at pH 9 also caused deregulation of glycolytic metabolite levels in wild-type cells, it was not possible to assess the effect of the restored intracellular pH on long-term recovery of growth on glucose in *tps1*Δ cells under these experimental conditions.

A possible alternative explanation for the partial restoration of glycolytic metabolite levels when glucose was added together with 200 mM NH_4_Cl at pH 9, as opposed to those at pH 9 without NH_4_Cl, is that the high NH_4_Cl concentration reduced glucose uptake to such an extent that glucose influx in glycolysis became limiting and the absence of the hexokinase Tre6P control therefore was no longer relevant. It is well established that a reduction of glucose transport can effectively lower glycolytic flux in S. cerevisiae (52). This is likely also the reason why *tps1*Δ cells can still grow at low glucose concentrations of 0.5 to 2 mM (Fig. 1b). This hypothesis could explain the strong reduction in sugar phosphate accumulation in *tps1*Δ cells (Fig. 6c and d), but it is more difficult to reconcile with the strong elevation of Fru1,6bisP levels in wild-type cells when glucose was added together with 200 mM NH_4_Cl at pH 9 (Fig. 6d). To evaluate this alternative explanation more rigorously, we first measured the glucose uptake rate at pH 9 in the absence and presence of 200 mM NH_4_Cl. This revealed a rather modest drop in glucose uptake activity of 30% to 35% (Fig. 7a). To assess the relevance of such a drop in the glucose uptake rate for the deregulation of glycolytic metabolite levels in *tps1*Δ cells, we constructed a *tps1*Δ strain with the additional deletion of five *HXT* glucose carrier genes, resulting in a strain with only low-affinity glucose uptake. This strain displayed a much higher reduction (65%) in glucose uptake but still was not able to grow on 100 mM glucose. Consistently, glucose-induced hyperaccumulation of sugar phosphates in that strain was only delayed and not prevented (Fig. 7c and d). These results contradict the alternative explanation that the experimental conditions used to prevent the persistent intracellular pH drop in *tps1*Δ cells after glucose addition caused a reduction in glucose influx in glycolysis high enough to weaken the deregulation of glycolysis as significantly as we observed. Hence, we are bound to conclude that the aberrant pH_i_ profile in *tps1*Δ cells after glucose addition is likely a major contributor to the deregulation of glycolysis, reducing GAPDH activity to such an extent that Fru1,6bisP starts to accumulate to very high levels, triggering ROS formation and apoptosis by hyperactivation of the Ras-cAMP-PKA pathway (12).

Our results support the importance of cytosolic pH for metabolic regulation. Under normal physiological conditions, the intracellular pH acidifies rather gradually toward the end of fermentation or drops suddenly in response to acute glucose starvation (53). A study on single-cell glycolytic oscillations showed that fluctuations in cytosolic pH as small as 0.1 to 0.3 can affect NADH concentrations (54). It might also affect GAPDH activity given its high sensitivity to changes in pH. A strong reduction in GAPDH activity has been described in the case of acute oxidative stress. The catalytic site of GAPDH is known to be very sensitive to oxidation, causing glycolytic flux to be rerouted to the pentose phosphate pathway for increased production of NADPH to combat ROS toxicity (31, 32). Interestingly, several conditions that are associated with increased ROS production, such as carbon starvation and the stationary phase (55), are associated with lowered cytosolic pH (53, 56).

Our work has also revealed novel regulatory effects caused by the deletion of *TPS1*. Although the allosteric inhibition of hexokinase activity by Tre6P provided a seemingly logical explanation for the overactive and persistent sugar phosphate accumulation and resulting glycolytic deregulation in *tps1*Δ cells (18), multiple experimental results have been obtained indicating that Tps1 must exert additional controls on yeast metabolism (19, 21, 27, 29). For instance, the expression of Tre6P-insensitive hexokinase from Schizosaccharomyces pombe in S. cerevisiae does not cause glycolytic deregulation (21). It should also be pointed out that the deletion of *HXK2* has important consequences besides lowering hexokinase activity, which might contribute to the restoration of growth on glucose of the *tps1*Δ strain, such as the loss of the glucose-repressed state (57) and the mislocalization of Ras to the mitochondria (58). In addition, *tps1* mutants also show defects on media with respiratory carbon sources, i.e., in the absence of glucose, such as the change in regulatory properties of glycogen synthase (11) and the sporulation defect (1). By examining the contribution of the different Tdh isoenzymes of GAPDH in the *tps1*Δ growth defect, we unexpectedly discovered that the deletion of *TDH3* in a *tps1*Δ strain severely compromises growth on galactose, glycerol, and ethanol, whereas it produces no noticeable defect in a wild-type strain. This points to the existence of an unknown regulatory connection between Tps1 and GAPDH, since hexokinase activity is not required for growth on galactose, glycerol, and ethanol and the absence of its Tre6P control therefore would not be expected to have any relevance. On the other hand, GAPDH activity is required for growth on galactose, glycerol, and ethanol, and since only the deletion of *TDH3* caused this growth problem, the results indicate that Tps1 is required in some way for the maintenance of proper Tdh1 and Tdh2 activity in the absence of Tdh3. Future work will have to reveal the precise underlying mechanism of this novel connection between Tps1 and GAPDH.

### Conclusions.

Our work reveals that the apparent bottleneck in glycolysis at the level of GAPDH after the addition of glucose to cells of the *tps1*Δ mutant is, at least in part, due to the combination of the unusually high pH optimum of GAPDH and the persistent intracellular acidification caused by the deregulation of glycolysis. Counteraction of the intracellular acidification strongly reduced Fru1,6bisP hyperaccumulation and restored extracellular medium acidification, consistent with partial restoration of glycolytic flux. Our work provides further evidence for the importance of intracellular pH regulation in the control of metabolism. In addition, we provide evidence for novel regulatory defects caused by *tps1*Δ in combination with *tdh3*Δ, which are independent of the Tps1 control on hexokinase activity.

## MATERIALS AND METHODS

### Yeast strain and plasmid overview.

All yeast strains used in this work share the same W303 genetic background. Strains and plasmids used and generated in this study are listed in Tables 1 and 2, respectively. For gene deletions, coding sequences of antibiotic resistance genes or auxotrophic markers were PCR amplified using primers with tails designed to be homologous to the 50 bp directly upstream or downstream of the gene of interest. Genes were deleted by means of homologous recombination. Both linear and plasmid DNA were introduced through the lithium acetate heat shock protocol at 42°C reported by Gietz and Schiestl (59). For transformations with antibiotic selection, cells were recovered in liquid medium without selection for 4 h prior to plating on medium with the antibiotic. For transformations with auxotrophic selection, cells were immediately plated after heat shock.

**TABLE 1:** List of the strains used and generated in this work

Strain/main genotype	Complete genotype	Source or reference
W303-1A (JT 9019)	*MAT***a** *leu2-3,112 trp1-1 can1-100 ura3-1 ade2-1 his3-11,15 GAL SUC mal*	63
*tps1*Δ (JT 9020)	*MAT***a** *leu2-3,112 trp1-1 can1-100 ura3-1 ade2-1 his3-11,15 GAL SUC mal tps1*::*TRP1*	13
*tps1*Δ *tdh1*Δ	*MAT***a** *leu2-3,112 trp1-1 can1-100 ura3-1 ade2-1 his3-11,15 GAL SUC mal tps1*::*TRP1 tdh1*::*KanMX*	This study
*tps1*Δ *tdh2*Δ	*MAT***a** *leu2-3,112 trp1-1 can1-100 ura3-1 ade2-1 his3-11,15 GAL SUC mal tps1*::*TRP1 tdh2*::*KanMX*	This study
*tps1*Δ *tdh3*Δ	*MAT***a** *leu2-3,112 trp1-1 can1-100 ura3-1 ade2-1 his3-11,15 GAL SUC mal tps1*::*TRP1 tdh3*::*KanMX*	This study
*tps1*Δ *hxk2*Δ	*MAT***a** *leu2-3,112 trp1-1 can1-100 ura3-1 ade2-1 his3-11,15 GAL SUC mal tps1*::*TRP1 hxk2*::*LEU2*	This study
*tps1*Δ *snf1*Δ	*MAT***a** *leu2-3,112 trp1-1 can1-100 ura3-1 ade2-1 his3-11,15 GAL SUC mal tps1*::*TRP1 snf1*::*HIS3*	25
*hxt6,7,2,4,5*Δ	*MAT***a** *leu2-3,112 trp1-1 can1-100 ura3-1 ade2-1 his3-11,15 GAL SUC mal HXT6,7*::*looped HXT2*::*looped HXT4*::*NatMX HXT5*::*KanMX*	This study
*tps1*Δ *hxt6,7,2,4,5*Δ	*MAT***a** *leu2-3,112 trp1-1 can1-100 ura3-1 ade2-1 his3-11,15 GAL SUC mal tps1*::*HphMX HXT6,7*::*looped HXT2*::*looped HXT4*::*NatMX HXT5*::*KanMX*	This study

**TABLE 2:** List of the plasmids used and generated in this work

Plasmid backbone	Vector element	Selection marker	Insert	Source or reference
p426	Multicopy 2 μ, *TEF1* promotor	*KanMX*	None	A. Claes (MCB lab)
			*TDH2*	T. Nicolaï (MCB lab)
			*TDH3*	T. Nicolaï (MCB lab)
pYES2	Multicopy 2 μ, *ACT1* promotor	*URA3*	pHluorin	53

### General growth conditions.

Yeast cells were grown in either rich or minimal medium. For rich medium, cells were grown in YP (1% [wt/vol] yeast extract, 2% [wt/vol] bacteriological peptone) supplemented with 100 mg/liter adenine. In case of minimal medium, cells were grown in complete synthetic medium (MP biomedicals) containing 0.17% (wt/vol) yeast nitrogen base without amino acids and ammonium sulfate (Oxoid), supplemented with 0.5% (wt/vol) ammonium sulfate (Sigma-Aldrich) and 100 mg/liter adenine. The same medium with the appropriate amount of essential amino acid(s) was used for growth of auxotrophic strains. As a carbon source, either 100 mM glucose, 100 mM galactose, 220 mM ethanol, or 325 mM glycerol was added to the growth media. The pH was adjusted to 5.5 for liquid medium and 6.5 for solid medium (2% agar) with KOH. Strains were grown at 30°C under continuous shaking for liquid cultures (180 rpm). For assay purposes, cells were typically pregrown overnight and inoculated the next day in fresh medium. Cells were harvested in the exponential phase unless mentioned otherwise.

### Assessment of cell proliferation.

**(i) Spot tests.** For spot tests of growth capacity, cells were pregrown overnight on 3% glycerol in rich medium until the late-exponential early stationary phase. For cells harboring the p426 overexpression plasmid, 100 mg/liter Geneticin was supplemented in both liquid and solid media to retain the plasmid. Next, cells were washed and spotted at a 5-fold serial dilution (starting from optical density at 600 nm [OD_600_] = 0.5) on freshly poured solid media with the carbon source of interest. Pictures were taken after 2 to 3 days of incubation at 30°C.

**(ii) Growth curves.** To determine growth curves, cells from an overnight preculture on galactose were harvested, washed, and resuspended to an OD_600_ of 0.1 in a 96-well plate. OD_595_ was measured every 30 min for 48 h using the Thermo Scientific Multiskan FC microplate photometer. Plates were shaken every 10 min for 1 min at medium speed to keep cells in suspension.

### Metabolite measurements.

Metabolites were extracted and measured as described previously Peeters et al. (12). General procedures will be mentioned in short. All auxiliary enzymes were purchased from Sigma-Aldrich.

**(i) Sample collection.** Cells for metabolite measurements were cultured in complete synthetic medium with 2% galactose (unless stated otherwise) to the exponential phase, harvested by centrifugation, and washed twice with 25 mM morpholineethanesulfonic acid (MES), pH 6. Cells were resuspended at a concentration of 75 mg (wet weight)/ml in complete synthetic medium with no added sugar and incubated at 30°C for 30 min of acclimatization. For metabolite determination after glucose or galactose addition, the sugars were added to a final concentration of 100 mM. Samples were taken by quenching cell suspensions in 60% methanol at −40°C (60). Quenched cells were centrifuged to remove the supernatant, after which, the pellet was resuspended in 0.5 ml 1 M HClO_4_ and transferred to a screw-cap tube. After mechanical lysis, an additional 0.5 ml of 1 M HClO_4_ was added, after which, the samples were stored at −20°C.

**(ii) Sample processing.** Thawed cell lysates were spun down at high speed and supernatants were collected. From this point on, cell lysates needed to undergo a process of neutralization. First, 50 μl of 5 M K_2_CO_3_ was added to 250 μl cell lysate together with 10 μl thymol blue (0.025%) to monitor the pH visually. Samples were allowed to degas on ice for 15 min. Next, samples were spun down at high speed, and 200 μl supernatant was added to 100 μl 1 M HCl and 10 μl Tris-HCl (pH 7.5). Samples were stored at −20°C.

**(iii) Metabolite measurements.** Metabolite concentrations were determined by endpoint measurement of the absorption of NADH or NADPH at 340 nm through coupled enzymatic reactions. The increase or decrease of absorption correlates linearly with the metabolite’s concentration and was calculated by applying Lambert’s law. The general reaction buffer was composed of 50 μl sample incubated with 150 μl 100 mM Tris-HCl, pH 7.5. Other components were added to the reaction buffer depending on the metabolite to be measured. For measurement of Glu6P, basal absorption was measured at 340 nm from samples added to reaction buffer supplemented with 0.8 mg/ml NADP^+^ and 10 mM MgCl_2_. After addition of Glu6P dehydrogenase at a final concentration of 50 μg/ml, the difference in OD_340_ values was used to calculate the absolute concentration of Glu6P. For measurement of ATP, the stabilized absorption spectrum of the Glu6P samples was taken as the new basal level. Next, hexokinase was added to a final concentration of 100 μg/ml together with 0.5 mM glucose. Stabilized spectra were used for calculating the corresponding ATP concentration.

For measurement of Fru1,6bisP, the general reaction buffer was supplemented with 0.8 μg/ml NADH, 25 μg/ml triosephosphate isomerase, and 25 μg/ml glycerol-3-phosphate dehydrogenase. After measuring basal absorption, 200 μg/ml aldolase was added, and the difference in OD_340_ values was used to calculate Fru1,6bisP levels. To express metabolite levels in terms of cytosolic concentration, an intracellular volume of 12 μl/mg protein was assumed.

### Determination of *in situ* GAPDH activity.

**(i) Preparation of permeabilized spheroplasts.** The protocol for preparation of spheroplasts was adapted from previous reports (29). Strains were grown on 2% galactose in rich medium to the exponential phase. Cells were harvested, washed with 25 mM MES (pH 6), and resuspended at a concentration of 200 mg (wet weight)/ml in digestion buffer containing 1.2 M sorbitol, 60 mM potassium phosphate buffer (pH 7.5) (K_2_HPO_4_ plus KH_2_PO_4_), 1 mM EDTA (pH 8), 10 μl/ml β-mercaptoethanol, and 100 U/ml lyticase. Cell wall digestion at 30°C was followed by sampling the cell suspension for decrease in OD_600_ when treated with 5% SDS. When the OD_600_ dropped to around 80%, spheroplasts were considered appropriate for use, and lyticase digestion was stopped by addition of four volumes ice-cold 1.2 M sorbitol buffer. Spheroplasts were washed twice with ice-cold 1.2 M sorbitol and resuspended in spheroplast buffer (1.2 M sorbitol, 0.75 mM EDTA, 2 mM MgSO_4_, 1.8 mM NaCl, and 10 mM potassium phosphate buffer, pH 6.8). Protein concentration was measured in order to dilute the spheroplast suspension to a final protein concentration of 2 mg/ml. To permeabilize the spheroplasts, they were incubated for 10 min at 30°C with 20 μl/ml nystatin prior to measuring enzyme activity.

**(ii) Determination of specific GAPDH activity.** To measure GAPDH activity using permeabilized spheroplasts, the reaction buffer was composed of spheroplast buffer, 2 mM GA3P, 4 mM NAD^+^, and 20 mM Tris-HCl. The pH of the reaction buffer was adjusted with HCl/KOH to obtain the desired pH values. The influence of Tre6P and Fru1,6bisP on GAPDH activity was examined at pH 6.8. The reaction was initiated by adding 5 μl spheroplast suspension (2 mg/ml) to 145 μl reaction buffer. The initial reaction velocity was calculated from the measured linear increase of absorbance at 340 nm using the Synergy H1 Hybrid reader. Specific GAPDH activity was expressed as nanomoles per minute per milligram protein.

### Determination of cytosolic pH.

For determination of intracellular pH, the pHluorin plasmid was transformed into strains of interest. The cells were grown in uracil dropout medium to retain the plasmid but with LoFlo formulation (ForMedium) to minimize background fluorescence. After reaching the exponential phase on 2% galactose, cells were harvested, washed, and resuspended in LoFlo medium at pH 5.5. Emission spectra were monitored at 510 nm after excitation at the wavelengths of 485 nm (510^Ex: 485 nm^) and 390 nm (510^Ex: 390 nm^). The ratio (510^Ex: 485 nm^/510^Ex: 390 nm^) was used to extrapolate the corresponding pH value using a calibration curve obtained by incubation of cell suspensions in calibration buffer at various pHs (25 mM MES, 25 mM HEPES, 25 mM KCl, 25 mM NaCl, 0.1 M ammonium acetate, 5 mM NaN_3_, and 0.18 mM 2-deoxyglucose). For experiments involving addition of NH_4_Cl, LoFlo medium was buffered with 200 mM Tris-HCl at the desired pH. Prior to treatment, baseline fluorescence was measured for 5 min. Using an integrated dispensing system that allows for quasisimultaneous pipetting and measurement (Thermo Scientific Fluoroskan Ascent FL with 390/510 and 485/510 filter sets), glucose and NH_4_Cl at final concentrations of 100 mM and 200 mM, respectively, were added at time point zero, after which, fluorescence was measured for the desired time periods. Per strain, emission spectra were measured for at least 9 technical repeats to consider the variability between experiments.

### Determination of glucose uptake.

Transport of [U-^14^C]glucose was measured in accordance with previous studies by Reifenberger et al. (61) and Özcan et al. (62). In short, cells were grown in complete synthetic medium with 2% galactose. After reaching the exponential phase, cells were washed once with ice-cold 25 mM MES (pH 6), weighed, and resuspended in complete synthetic medium to a final concentration of 45 mg (wet weight)/ml. The cell suspension was dispensed into test tubes on ice and through a rotating schedule, each sample was temperature acclimated for 10 min at 30°C prior to the measurement of glucose uptake. For uptake of 100 mM glucose, an adequate amount of [U-^14^C]glucose was added to obtain after 10 s a response of at least 1,000 counts per min to create a sufficient signal-to-noise ratio. At time point zero, labeled glucose was added to the cells, and the suspension was quickly vortexed and quenched after 10 s with 5 ml ice-cold H_2_O, after which, the cell suspension was immediately filtered over a glass microfiber filter (Whatman GF/C). Cells were additionally washed on the filter two more times with water, after which, the loaded filter was transferred to a scintillation vial containing 3 ml liquid scintillation cocktail (Ultima-Flo M; PerkinElmer). To account for background signal originating from filter-attached radiolabeled glucose, three blank measurements per strain were included, for which cells where quenched prior to adding the labeled glucose. In experiments in which the effect of NH_4_Cl was assessed (Fig. 7a), Tris-HCl (pH 9) was added to the cells 5 s prior to glucose and NH_4_Cl addition. The final concentration of Tris-HCl and NH_4_Cl was 200 mM. Scintillation counting was performed using the Hidex 300 SL.
