# Supplementary figures and images for: Aberrant Intracellular pH Regulation Limiting Glyceraldehyde-3-Phosphate Dehydrogenase Activity in the Glucose-Sensitive Yeast tps1Δ Mutant
Source: mBio. 2020 Oct 27;11(5):e02199-20. doi: 10.1128/mBio.02199-20 (PMC7593968; doi:10.1128/mBio.02199-20)

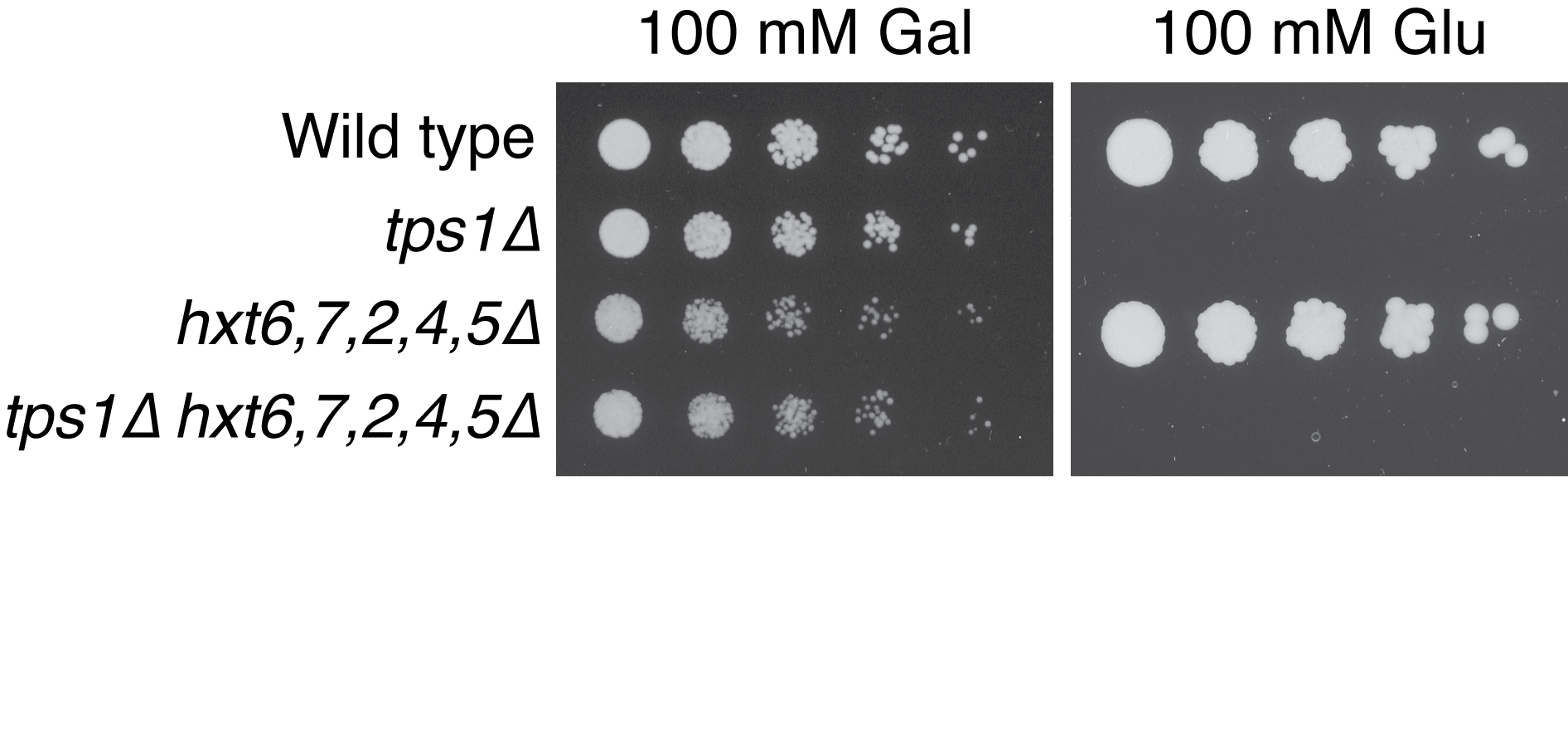

Supplement: FIG S3 [file mBio.02199-20-sf003.tif]
